# Diagnostic Value of Oral Provocation Tests in Drug Hypersensitivity Reactions Induced by Nonsteroidal Anti-Inflammatory Drugs and Paracetamol

**DOI:** 10.3390/diagnostics12123074

**Published:** 2022-12-07

**Authors:** Iwona Popiolek, Magdalena Blasiak, Aleksandra Kozak, Ewelina Pietak, Malgorzata Bulanda, Grzegorz Porebski

**Affiliations:** 1 Department of Toxicology and Environmental Diseases, Jagiellonian University Medical College, Jakubowskiego 2, 30-688 Krakow, Poland; 2 Department of Clinical and Environmental Allergology, Jagiellonian University Medical College, sw. Anny 12, 31-008 Krakow, Poland; 3 Department of Clinical and Environmental Allergology, Jagiellonian University Medical College, Botaniczna 3, 31-503 Krakow, Poland

**Keywords:** acetaminophen, adverse drug reactions, drug hypersensitivity, drug safety, immediate drug hypersensitivity reactions, negative predictive value, nonsteroidal anti-inflammatory drugs, NSAIDs, oral provocation test, paracetamol

## Abstract

Oral drug provocation tests (DPT) are the basic diagnostic tool for the detection of hypersensitivity to non-opioid analgesics and for selecting a safe alternative for a patient. They are of great practical importance due to their common use, but the data on the follow-up of patients after negative DPT are still very scarce. We examined the further fate of 164 such adult patients after negative NSAID or paracetamol tests and analyzed which excipients in the studied drugs they could be exposed to after the diagnostic workup. A structured medical interview was performed 32.9 months (mean) after the provocation tests. Of the 164 patients, 131 (79.9%) retook the tested drug and 12 developed another hypersensitivity reaction, giving the estimated negative predictive value of 90.8%. These reactions were induced by acetylsalicylic acid, paracetamol, meloxicam, and diclofenac, and were clinically similar to the initial ones (most commonly urticaria and angioedema). There are 93 generics of these drugs on the local market, containing a total of 33 excipients for which hypersensitivity reactions have been reported. All available generics contain such excipients. Thirty-one patients (20.1%) did not take the previously tested drug again, most often because it was not needed or because they were afraid of another reaction. DPT with analgesics has a high diagnostic performance. A minority of patients had relapsed after reexposure. One of the underestimated reasons for this may be drug excipients provoking a reaction, so it is advisable to use exactly the same medical product that has been negatively tested. Many patients avoid reexposure to a given drug, despite negative tests, therefore very reliable patient education in connection with DPT is highly needed.

## 1. Introduction

Drug hypersensitivity reactions (DHR) belong to type B adverse drug reactions, which are pharmacologically unpredictable and clinically resemble allergic symptoms [1]. They have a substantial impact on both everyday clinical practice and the health care system on a global scale. DHR can be life-threatening and affects up to 7% of the general population [2] and up to 5% of hospitalized patients [3]. Nonsteroidal anti-inflammatory drugs (NSAID) and paracetamol (PRC) are among the most commonly prescribed drugs and are also easily obtained over-the-counter [4,5]. Simultaneously, they are, besides antibiotics, the leading group of drugs responsible for drug hypersensitivity reactions [6,7,8,9,10].

NSAID differs in chemical structure but have the same pharmacological properties dependent on cyclooxygenase 1 (COX-1) and/or COX-2 inhibition [11]. Most NSAID-induced DHR are related to this mechanism of action, which interferes with arachidonic acid metabolism and leads to leukotriene overproduction and blockage of prostaglandin synthesis, and, consequently, to the development of hypersensitivity symptoms [11,12]. PRC hypersensitivity reactions are often considered in the context of NSAID hypersensitivity because, on the one hand, it is usually taken into account as a possible alternative painkiller in patients with hypersensitivity to NSAID [13] but, on the other hand, the overall cross-reaction rates with PRC in these patients reach 24.8% [14].

Depending on main symptoms, timing, underlying disease, and background mechanisms, NSAID-hypersensitive reactions have been divided into a few types: (i) NSAID-exacerbated respiratory disease (NERD) with bronchial asthma/rhinosinusitis, (ii) NSAID-exacerbated cutaneous disease (NECD) with chronic urticaria, and (iii) NSAID-induced urticaria-angioedema (NIUA) without underlying chronic diseases. These types of reactions show cross-reactivity between NSAID, whereas the other two types do not: (iv) Single-NSAID-induced urticaria/angioedema or anaphylaxis (SNIUAA) and (v) Single-NSAID-induced delayed reactions (SNIDR) [13]. These last two types are considered to be mediated by IgE and T cells, respectively [15,16].

Because non-opioid painkiller drugs (NSAID and PRC) are widely used for many medical conditions, patients suspected of hypersensitivity reactions to them are in high need of offering a safe alternative drug [6]. Weak COX-1 inhibitors, preferential or selective COX-2 inhibitors, are supposed to be often well tolerated by NSAID-hypersensitive patients [17], but this cannot be taken for granted [14,18]. Due to the shortcomings of in vitro methods and limitations in the use of skin tests, drug provocation tests (DPT) are in most cases the method of choice to confirm hypersensitivity to a given drug or to verify tolerance to alternatives [6].

As a matter of fact, DPT is recommended as the gold standard for diagnosing NSAID hypersensitivity [1,13,19,20]. The test consists of the controlled administration of a drug suspected of inducing a hypersensitivity reaction or a drug with analogous properties that may serve as an alternative for treatment. Because severe hypersensitivity symptoms, which were observed during index reaction, may be reproduced during provocation, DPT should be performed under hospital surveillance [10]. Negative DPT allows the ability to rule out drug hypersensitivity or to indicate a safe alternative drug. This piece of information is crucial for further patient management because it allows one to safely prescribe a drug after a negative diagnostic workup. However, it may happen that reexposure to this drug produces hypersensitive symptoms again [21]. Furthermore, some patients may be afraid to use the drug despite negative DPT [22]. However, data on the follow-up of patients after negative DPT are still very scarce [23] and the evaluation of predictive values of provocation tests in NSAID hypersensitivity is still recognized as an unmet need [13].

In the presented study, we analyzed a cohort of patients suspected of NSAID hypersensitivity who underwent DPT with a suspected or an alternative drug. The main objectives of the study were (i) to determine the frequency of false negative DPT, which corresponds to a negative predictive value (NPV) of the test, and (ii) to assess patients’ attitude to taking non-opioid painkiller drugs again after allergologic workup. We also analyzed (i) differences in culprit drugs and clinical patterns between initial hypersensitivity reactions and reactions after reexposure, (ii) differences in clinical characteristics and demographic features between patients with true negative and false negative DPT, as well as (iii) excipients present in generic drugs, which may be responsible for hypersensitivity reactions, and this way justify some false negative DPT.

Regarding the main objective of our study, namely to determine the NPV in the study group and the percentage of patients avoiding the selected drug, findings from this study revealed that an NPV in our investigated cohort reached 91%, which is a satisfactory result, but still leaves space for unexpected reactions after reexposure to a drug. A significant number of tested patients (20%) did not dare take a tested drug again. This indicates an urgent need to educate patients about the benefits of DPT before performing this time-consuming and costly diagnostic workup. Finally, many of the excipients that are present in drugs showing false negative DPT are known to induce allergic reactions. Those excipients are present only in the selected generics containing the same active substance; therefore, in a real-life setting, they may induce hypersensitive reactions independently of NSAID.

## 2. Materials and Methods

### 2.1. Study Group

We investigated all consecutive patients who were referred to our department from January 2016 to December 2020 by their primary physicians due to reactions suggesting hypersensitivity induced by NSAID or PRC and who were negatively tested against a drug in question. Routine diagnostic workup involved anamnesis based on the standardized ENDA questionnaire [24], skin tests with common environmental allergens and drugs, and DPT with a drug in question. The evaluation was performed at least 6 weeks after the clinical symptoms of the hypersensitivity reaction resolved.

The specific criteria for patient enrollment were the following: adults; clinical history suggestive of NSAID or PRC hypersensitivity with NERD, NIUA, or SNIUAA phenotypes defined based on anamnesis according to recommendations [13]; and negative diagnostic workup including DPT. We excluded patients with hypersensitivity to NSAID or PRC confirmed during the testing, patients who did not undergo DPT due to contraindications (specified below), patients with a diagnosis of chronic urticaria (corresponding to the NECD phenotype) and with single-NSAID-induced delayed reactions; and the patients with a clinical history not compatible with the drug hypersensitivity reaction (e.g., symptoms and signs persisting despite discontinuation of drug intake).

### 2.2. Diagnostic Tests

Skin prick tests and intradermal tests with the drugs in question were performed as recommended [25,26] in patients demonstrating the SNIUAA phenotype, which may have an IgE-dependent mechanism [13]. Individual atopic status was screened by skin prick tests against the panel of aeroallergens adapted to exposition in the local area, which consisted of seasonal (birch, alder, hazel, mixed grass pollens, mugwort, Alternaria alternate, and Cladosporium album) and perennial allergens (Dermatophagoides pteronyssinus, Dermatophagoides farine, and cat and dog fur) (Allergopharma, Reinbek, Germany). The tests were carried out and interpreted according to published standards [27]. Medications that may suppress the skin test were withdrawn before testing with aeroallergens and drugs for the required time [25,26,27].

DPT were performed according to protocols recommended by the European Academy of Allergy and Clinical Immunology/European Network for Drug Allergy [20,28,29]. Briefly, we applied a single-blinded design with a placebo administered on day 1. The next day, a patient was given a tested drug orally that was suspected of causing the initial hypersensitivity reaction or selected as a potentially safe alternative. For aspirin, a four-step approach was performed (71, 117, 312, and, optionally, 500 mg of aspirin given every 60 to 90 min), as previously described [28]. Other drugs were administered in three to four steps in 1.5–2 h intervals from 1/10 of the single dose up to the usual daily dose [13]. 

The DPT were performed under strict hospital surveillance by the staff with resuscitation support. Patients were closely monitored during the test and up to 6 h after the last dose. The tests were considered negative if there were no objective symptoms and signs of hypersensitivity. Regarding the parameters of lung function, the decrease in forced expiratory volume in 1 s (FEV1) or peak expiratory flow (PEF) ≥ 20% from baseline were considered significant [28]. Any medications that might inhibit a response in DPT were stopped before the tests according to recommendations [20,28,29]. We excluded from DPT patients with known contraindications: history of drug-induced severe cutaneous adverse drug reactions (acute generalized exanthematous pustulosis, Stevens–Johnson syndrome/toxic epidermal necrolysis, and drug reaction with eosinophilia and systemic symptoms), severe organ-specific hypersensitivity reactions (e.g., nephritis, hepatitis, and pneumonitis), or any severe disease, cardiovascular disease under beta-blockers, and pregnancy [13,20,28,29].

### 2.3. Follow-Up

Patients were contacted at least 9 months after the completed diagnostic workup by phone or at the regular control visit in our outpatient clinic. In the structured medical interview, we asked the patients about the intake of analgesics after a negative DPT, how they were tolerated, or reasons for avoidance. The questions included the following: (i) has the patient taken the previously suspected analgesic and/or another one since she or he underwent a negative diagnostic workup involving DPT?; (ii) if yes, did any hypersensitive reactions occur?; (iii) if not, what was the reason for avoidance?; and (iv) if a reaction has occurred, what were the symptoms, and what drug was taken?

**Figure 1:**
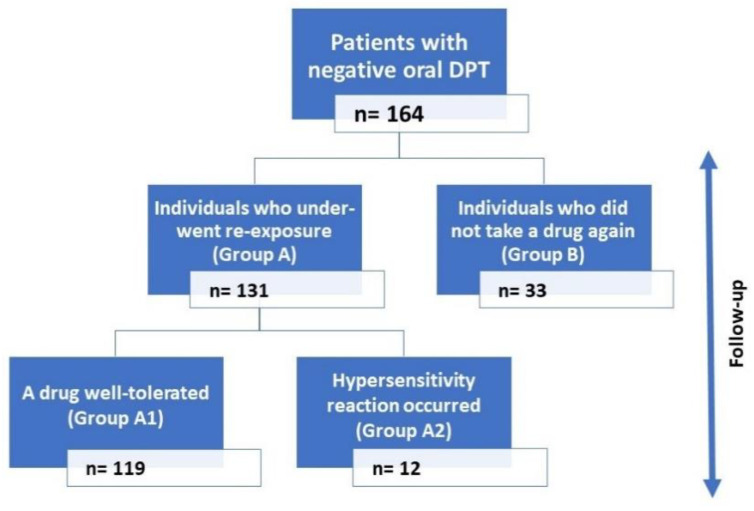
The results of the follow-up interview regarding reexposure to the drugs.

According to the data obtained, the patients were divided into group A (who took a drug after negative DPT) and group B (who did not take a drug after negative DPT). Two subgroups were distinguished in group A: subgroup A1 (the drugs taken were well tolerated) and subgroup A2 (the drugs taken induced a hypersensitive reaction). In further analysis, we compared groups A and B and subgroups A1 and A2 with regard to the clinical characteristic and the drugs involved. The general framework of the study is shown in Figure 1.

### 2.4. Identification of Excipients in Generic Drugs of Interest

To answer the question, of whether there is the possibility that excipients, but not the active substances by themselves, are inducing hypersensitivity reactions under reexposure, we performed an approach as follows. After negative oral provocation tests, patients could use any drugs with the tested active substance, therefore, we searched for all generics with the active substances that were identified in patients from group A2 (hypersensitivity after reexposure). For this purpose, we used the lists available on the website of *the Office for Registration of Medicinal Products, Medical Devices and Biocidal Products*, which presents all the medicinal products available in Poland [30].

In the next step, we analyzed Summaries of Product Characteristics [30] for all the medicinal products found. Because Summaries of Product Characteristics (SmPC) are documents created in a standardized form in accordance with the rules governing medicinal products, in Supplementary Materials of SmPC one can always find a “list of excipients” that a given product contains. We retrieved data from Supplementary Materials of SmPC and identified all excipients which were present in the drugs of interest. Finally, we performed a structured search on the Pubmed electronic database for reports on hypersensitivity/allergic reactions induced by the previously pinpointed excipients. The search strategy: ‘substance’ AND (‘hypersensitivity’ OR ‘allergy’) AND ‘case reports’ was applied without limitation on the publication date for every single excipient.

### 2.5. Statistical Analysis

For all included patients, we recorded information on demographic characteristics, clinical features (including phenotype of initial reactions: NERD, NIUA, or SNIUA), the analgesic drugs involved, and the results of the diagnostic tests. Nominal data were provided as numbers with absolute or relative frequencies. Continuous variables were expressed as means with standard deviation. The NPV of oral DPT was calculated as the ratio of patients who tolerate reexposure (truly negative results) to all patients who had negative test results. To compare the groups, we used the Mann–Whitney U test, and the chi-square test or Fisher’s exact test, where appropriate. A *p*-value less than 0.05 was considered statistically significant. Statistical analysis was performed using Statistica (data analysis software system), version 13, TIBCO Software Inc., 2017, Tulsa, OK, USA.

## 3. Results

### 3.1. General Characteristic of the Study Group

In the study, we analyzed patients who underwent oral DPT with analgesics (NSAID or PRC) with negative results. The study group consisted of 164 subjects with a mean age of 52.9 ± 16.1 years (range: 18–84 years), 133 women (81%), and 31 men (19%). We found that 89 of them represented the NIUA phenotype, 69 individuals the SNIUAA phenotype, and the other 6 the NERD phenotype. The mean time between the challenge test and the interview on reexposure was 32.9 ± 18.0 months. Skin prick tests with seasonal and perennial aeroallergens were positive in 23% and 27.8% of the tested individuals, respectively. All skin prick tests and intradermal tests with drugs were negative. Drugs suspected of causing an initial hypersensitivity reaction included: NSAID without specifying a distinct drug in medical history or more than one drug that induced reactions in the past (*n* = 89); acetylsalicylic acid, ASA (*n* = 25); ibuprofen, IBU (*n* = 9); diclofenac, DIC (*n* = 9); ketoprofen, KET (*n* = 8); metamizole, MET (*n* = 6); PRC (*n* = 6); naproxen, NAP (*n* = 5); nimesulide, NIM (*n* = 5); dexketoprofen, DKET (*n* = 1); and propyphenazone, PPP (*n* = 1). The results of the follow-up interview regarding reexposure to the drugs are shown in Figure 1.

### 3.2. Patients with False Negative Drug Provocation Tests (Group A2)

Among of the 164 followed patients, 131 had taken analgesic (NSAID or PRC) after a negative provocation test, and 12 of those 131 patients reported the next hypersensitivity reaction. Therefore, the NPV reached the 90.8% value. Among the drugs tested previously, the most hypersensitivity reactions after reexposure were observed after taking ASA, and the remaining cases were PRC, meloxicam (MEL), and DIC. Details of the drugs used and clinical signs related to initial and subsequent hypersensitivity reactions are presented in Table 1. The most common phenotype of an initial drug-induced reaction observed in group A2 was single-NSAID-induced urticaria/angioedema or anaphylaxis, and the other was NIUA (*n* = 3) and NERD (*n* = 2). The symptoms that developed after exposure corresponded in most cases with the initial phenotype of the reaction in a given patient (Table 1).

Patients who tolerated reexposure did not differ from those who responded to reexposure to drugs with symptoms of hypersensitivity in terms of age, the time span from the initial reaction to the interview, observed reaction phenotype, or atopy characteristics as expressed by positive skin tests with common aeroallergens (Table 2). The only demographic distinguishing characteristic was a statistically significantly higher number of women in group A2. Additionally, the comparative analysis of suspected drugs and drugs tested during DPT did not show any significant differences between group A1 and group A2. 

### 3.3. Patients Who Avoided Reexposure (Group B)

Thirty-three out of 164 patients (20%) did not use any NSAID or PRC despite the negative outcome of DPT. The absence of re-intake of the studied drugs lasted for 10.5 to 58.5 months (mean 30.9) at the moment of the interview. Reasons for avoiding the use of these drugs were in most cases that there was no need for such treatment (*n* = 17). In the next 15 cases, patients reported fear of the next drug hypersensitivity reaction as a reason for avoidance. One patient used opioids instead of non-opioid analgesics. Comparison of group A (reexposed to the drug) and group B (no reexposure) did not reveal any differences with respect to demographic or clinical characteristics, as well as the phenotype of hypersensitivity reaction, as shown in Table 3.

### 3.4. Excipients in Medicinal Products of Interest and Their Potential for Inducing Hypersensitivity Reactions

We took into consideration drugs that the patients reported as having induced hypersensitivity symptoms during reexposure, namely: acetylsalicylic acid, paracetamol, meloxicam, and diclofenac (Table 1). For these drugs, all generic medicinal products in tablet form were searched in the database of *the Polish Office for Registration of Medicinal Products, Medical Devices and Biocidal Products*. The following search results were received: 36 generics containing ASA (Table S1), 25 containing PRC (Table S2), 18 containing MEL (Table S3), and 14 with DIC (Table S4). The products found were in the form of plain tablets, enteric-coated tablets, effervescent tablets, coated tablets, prolonged-release tablets, and tablets orally disintegrating. Next, the names of all excipients were extracted from Summaries of Product Characteristics of each of these 93 generics (Tables S1 and S2).

Those excipient names were used in search of PubMed according to the strategy described in ‘the method section’ to disclose any publication concerning the potential for inducing hypersensitivity reactions by these substances. In this way, we were able to identify 33 excipients, for which different hypersensitivity reactions have already been described. In Table 4, we present the list of these substances together with a description of sample reports on hypersensitivity reactions to a given substance and the corresponding references. There were no medicinal products among ASA, PRC, MEL, or DIC generics without at least one of the 33 hypersensitivity-related excipients mentioned above (Tables S1–S4). Following the above search strategy, we also found some publications on the other 6 excipients (cellulose, polyvinyl alcohol, talc, simethicone, sodium citrate, and sodium bicarbonate). However, a closer analysis of the identified papers showed that these excipients were not associated with hypersensitivity reactions or that the reports were related to derivatives but not the excipients themselves.

## 4. Discussion

Diagnostic management of drug hypersensitivity includes medical history, physical examination, and provocation tests, which are of fundamental importance [4,13]. Skin tests and in vitro tests, although used in the diagnosis of various drug-induced reactions [75,76], are of limited use in the case of NSAIDs. Therefore, in this case, a precise assessment of the diagnostic value of provocation tests is extremely necessary for both doctors and patients. However, little is still known about the NPV of these tests, i.e., the test’s ability to deliver true negative results. To do this, it is necessary to evaluate the effects of the subjects’ reexposure to the drugs in question. So far, only single studies on beta-lactams [77,78], NSAIDs in the pediatric population [22,79] or adults [23,80], or various drugs [21,81] have been dedicated to the topic of NPV in provocation tests with drugs.

In our work, we analyzed the negative results of oral provocation tests with various NSAIDs and PRC, checking how many patients were exposed to the drugs tested and what their tolerance was. On this basis, we determined the NPV in the study group, which was 91%. This level was comparable to the results of other authors who examined patients after reactions to this group of drugs: 96–97% [22,23,79,80]. This provides new input for comparison of different populations that may use different generic drugs depending on a given region or country, updates our knowledge, checks for new trends in the clinical phenomena in this field, and also allows us to assess the quality of the diagnostics performed.

The most common clinical symptoms of the initial reactions in the study group (urticaria, angioedema, and rash) were similar to those observed by other authors [23], but our study managed to classify them according to the current phenotypes of NSAID hypersensitivity. Due to the potentially ambiguous result of the provocation and the assessment of subsequent reexposure, similar to Defrance et al. [23], we excluded patients with the NECD phenotype from the study group. An important factor in assessing the value of the test in the analyzed context is the time that elapsed from the challenge to the interview, during which time patients could undertake reexposure. In our study, it was 32.9 months (mean), compared to 33 months (median) in the study by Defrance et al. [23] and 5.1 years in the study by Jakić et al. [80]. The time span of 2–3 years seems to be favorable, while a longer delay may cause details of possible reactions to escape from the memory of the respondents during the survey. As expected, ASA was the drug most reported during initial reactions both in our study and in the other two studies discussed in the adult population [23,80].

In a group of 164 of our patients, in 12 (7%) reexposure to ASA, DIC, MEL, or PRC after negative DPT resulted in symptom recurrence. Similar percentages of reactions to drugs previously negatively tested were found in other studies, e.g., for NSAID from 2% to 5% [22,23,79], and for beta-lactams in the range of 0.5–11.4% [21,77]. The literature mentions many potential causes of the phenomenon of false negative provocation tests [1,2], ranging from (i) the importance of cofactors of drug hypersensitivity reactions (such as viral infections, exercise, and co-medication), which do not occur during scheduled provocation tests, (ii) induction of transient desensitization during DPT with gradually increasing doses of the study drug, until (iii) an independent cause of symptoms, e.g., viral infection, occurring coincidentally with the use of the suspect drug [77,80]. With regard to the IgE-dependent reaction, the mechanism of resensitization comes into play as well, which is revealed during subsequent reexposure to a given drug. However, for NSAID-induced reactions, this is less important. In general, these considerations are a hypothesis and evidence-based data is scarce.

The predominant symptoms of post-exposure skin hypersensitivity observed in group A2 corresponded to the initial phenotype of the reaction (Table 2). Only one patient with NERD (patient # 104) reported dyspnea, which also corresponds to his primary phenotype. The other patient with NERD (# 128) reported erythema several hours after reexposure, which is a nonspecific symptom. In the absence of medical verification, it could be an expression of an aggravation of the symptoms of the reaction by the patient. Importantly, none of the reactions after reexposure were life-threatening. Similar observations were made in this respect by other authors and they also emphasize this [23,82]. Some authors suggest that atopy predisposes the development of the NECD and NIUA phenotypes of hypersensitivity to NSAID [4], while others believe that the relationship between atopy and hypersensitivity to NSAID requires more data [6]. In our work, the characteristics of atopy, understood as the presence of positive skin prick tests with common aeroallergens, did not have any value differentiating between the studied subgroups (Table 2 and Table 3). However, the predominance of women in the entire study group was observed with a significantly higher percentage of women in the A1 group compared to A2. The importance of estrogens in allergic symptoms is not obvious, but their influence on mast cell activation is suggested, women also reported more allergic reactions, for example to food, and more adverse reactions to iodinated radiocontrast media [83]. Thus, in the group of DPT-negative patients with a history of hypersensitivity skin reactions, they may have a gender-related predilection for such reactions, regardless of NSAID hypersensitivity.

One of the important goals of our study was to evaluate the patient’s approach to the use of non-opioid analgesics after the diagnostic workup and after the selection of a safe drug. It turned out that 20% of the respondents did not use such a drug in a period of more than 2 years, despite the negative DPT. In other studies, these percentages differed significantly from each other. In the study by Defrance et al., it was 7%, while in the group evaluated by Bommarito, up to 47.4% of patients did not take the tested NSAID again [22]. Demoly et al. observed even higher percentages in patients tested with beta-lactams, of which more than 2/3 decided not to reexpose themselves to the drug [77].

It is difficult to find the reasons for this phenomenon in the specific demographic or clinical characteristics of the patients, as we did not observe any differences in this regard between the patients in groups A and B (Table 3). Our patients, similar to the group studied by Misirlioglu et al. [82], indicated that the lack of need for analgesics in the analyzed period was the most common reason. Given that it is a treatment commonly used in everyday life, it is expected that sooner or later there will be a need for analgesic/anti-inflammatory treatment in this group.

Another common reason for avoiding the drug tested was the fear of the next hypersensitivity reaction, which is consistent with the observations of other authors. In the group studied by Jakić et al., such a reason for avoiding reexposure was reported by up to 70.8% of patients who did not take the drug tested [80], and in the group of Misirlioglu et al. 45.2% [82]. Taking into account the already mentioned fact that even if the symptoms of hypersensitivity appear on reexposure, they are mild, such concerns of patients seem to be unjustified.

Certainly, help in improving this situation would be the increased education of patients already at the stage of qualifying for the diagnostic workup of drug hypersensitivity and the summary of recommendations and information on drugs selected for safe use in hospital discharge cards. This need is also indicated by other authors who deal with this problem [21,80]. This would probably avoid many costly and time-consuming procedures, which would not bring tangible benefits to patients anyway if the results of these procedures are not used. An additional complication for nonhealthcare professional patients is the large number of different generic drugs with the same active substance available on the market. We address this in the next part of this discussion.

The presence of additives and excipients in many non-opioid analgesics is relatively rarely analyzed as a possible cause of a reaction after taking a given drug during reexposure. Usually, after a negative DPT, the patient receives information that he can use the active substance, but, in his daily life, he can buy and take many generic drugs containing different excipients (Tables S1–S4). Many of them have allergenic potential and can cause hypersensitivity reactions, as shown in Table 4 [31,32,33,34,35,36,37,38,39,40,41,42,43,44,45,46,47,48,49,50,51,52,53,54,55,56,57,58,59,60,61,62,63,64,65,66,67,68,69,70,71,72,73,74]. An excellent illustration of this is the case series by Cox et al. [81]. It describes six patients allergic to polyethylene glycol, four of whom were originally suspected of being hypersensitive to NSAIDs, which was later ruled out by provocation tests. Excipients can be responsible for the induction of hypersensitivity with a wide variety of clinical manifestations, ranging from mild cutaneous manifestations to severe systemic reactions. The same substance can cause immediate and delayed hypersensitivity, e.g., hypromellose, for which cases of anaphylaxis [49] and contact allergy [48] are described. Hypersensitivity to excipients may also manifest as unusual reactions, e.g., oro-facial granulomatosis as a manifestation of hypersensitivity to sunset yellow [72].

In our group A2, symptoms reported after reexposure to ASA despite negative challenge were urticaria (5 persons), urticaria and erythema (1 person), angioedema (2 persons), dyspnea (1 person). In fact, many excipients in ASA generics (Table S1) can trigger such reactions: carmine [34], citric acid [38], cochineal red [39], glycine [44], hypromellose [48], macrogol [50], mannitol [52,53,54,55], polysorbate 80 [56,57], potato starch [63], and povidone [60,61,62]. Another patient developed urticaria after reexposure to MEL. Excipients in MEL generics (Table S3) can be responsible for an immediate immune reaction: potassium acesulfame [31], aspartame [33], citric acid [38], mannitol [52,53,54], and povidone [60,61,62].The next case of urticaria has been reported after reexposure to DIC. The following excipients found in generics of DIC have been reported to cause immediate reactions, among others, cochineal red [39], Hypromellose [22], mannitol [52,53,54,55], polysorbate 80 [56,57], and povidone [60,61,62] (Table S4). Finally, one of our patients, after reexposure to PRC, reported erythema several hours after exposure. Some examples of excipients in acetaminophen generics that can cause symptoms of hypersensitivity include alpha-tocopherol [32], colloidal silica [40], hypromellose [48], sorbitol [69], sodium benzo-ate [67], stearic acid [71], and titanium dioxide [74] (Table S2).

Individual generics may differ greatly in terms of the excipients they contain (e.g., Paracetamol Filofarm—Polyvinylpyrrolidone, Starch, Stearic acid vs. Paracetamol Aristo—Citric acid, Maltodextrin, Povidone, Sodium benzoate, Sorbitol; MeloxiMed vs. Mel—Colloidal silica Acesulfame potassium, Aspartame, Corn starch, Mannitol, Povidone; Abrea—Carmine, Colloidal silica, Macrogol, Polysorbate 80, Potato starch, Titanium dioxide vs. Aspirin—Corn starch; Voltaren SR 100—Cetyl alcohol, Colloidal silica, Hypromellose, Polysorbate 80, Povidone, Titanium dioxide vs. Olfen 75 SR—Hypromellose, and Titanium dioxide). Therefore, if an excipient is responsible for a hypersensitivity reaction, the same patient may tolerate one generic well and react with hypersensitivity symptoms to another.

**Figure 2:**
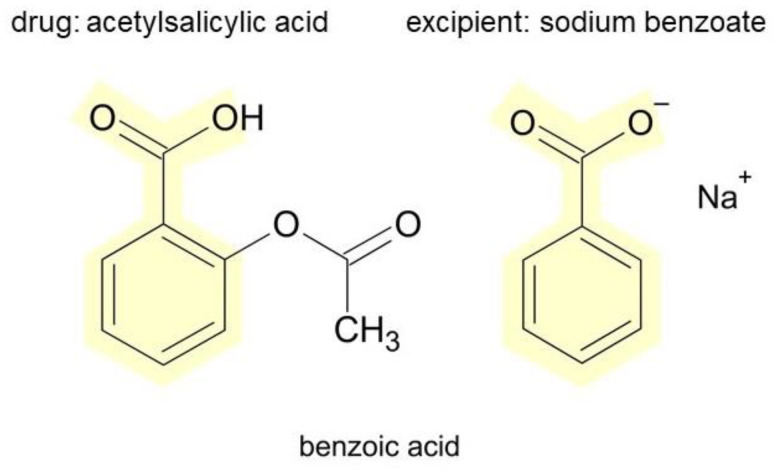
Comparison of the chemical formulas of ASA and its excipient sodium benzoate, which share the same benzoic acid structure (in yellow). Sketches were created with ChemSketch software, 1 February 2018. (Advanced Chemistry Development Inc., Ontario, Canada).

**Figure 3:**
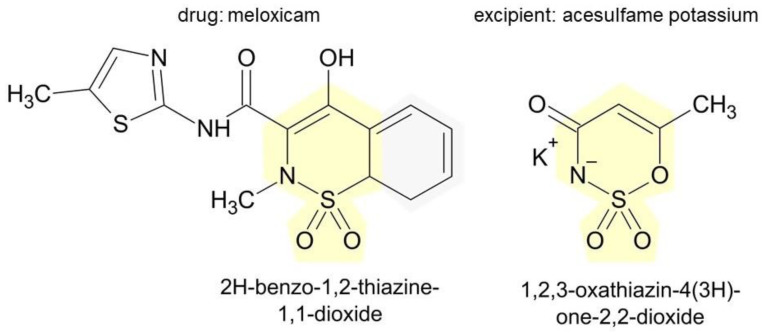
Comparison of the chemical formulas of MEL and its excipient acesulfame potassium, which share similar structures (in yellow). Sketches were created with ACD/ChemSketch software, 1 February 2018.

Comparison of the structures of individual drugs and excipients can provide further insight into this problem. Figure 2 and Figure 3 show the chemical formulas of the exemplary drugs that caused reexposure reactions and in parallel excipients that are present in some generics containing the given drug. Other examples are presented in Figure S1. It turns out that both groups of substances show structural similarities with each other, which may be related to the observed phenomena (e.g., potential cross-reactions). Of course, the more far-reaching conclusions are unjustified, but it seems that the variety of exogenous compounds to which we are exposed in industrialized societies is greatly underestimated. The division of many low-molecular compounds into drugs, cosmetics, preservatives, or dyes is formal, but does not necessarily reflect their impact on human health.

## 5. Conclusions

Our results show that oral provocation tests with analgesics have a high diagnostic performance. A minority of patients relapsed after reexposure to a given drug but these were never severe. Among the many potentially weakly understood causes of this phenomenon, one that is underestimated may be the various excipients found in generic medicines containing the same active substance. Therefore, it is reasonable to recommend that patients use exactly the same drug in their daily lives—the medical product that was used in their negative DPT. Many patients avoid reexposure to a given drug, despite negative tests, therefore another very important conclusion concerns the proper education of patients. The purpose and benefits of the proposed and performed diagnostics, supported by precise recommendations included in the discharge card and certificates for physicians of other specialties, should be thoroughly explained to them.

## Figures and Tables

**Table 1:** Initial and subsequent drug-induced reactions in group A2.

Patient’s Code	Suspected Drug	Phenotype of Initial Reaction	Tested Drug	Reaction Developed after Reexposure
18	NSAID	NIUA	ASA	angioedema (face, larynx)
124	NSAID	NIUA		urticaria
96	ASA	SNIUAA		urticaria
106	ASA	SNIUAA		urticaria and erythema
78	IBU	SNIUAA		angioedema (lips and eyelids)
9	PRC	SNIUAA		urticaria
47	NAP	SNIUAA		urticaria
68	KET	SNIUAA		urticaria
104	KET	NERD		dyspnoea
128	PRC	NERD	PRC	erythema
22	NSAID	NIUA	MEL	rush
70	DIC	SNIUAA	DIC	rush

ASA, acetylsalicylic acid; DIC, diclofenac; IBU, ibuprofen; KET, ketoprofen; MEL, meloxicam; MET, metamizole; NAP, naproxen; NERD, NSAID-exacerbated respiratory disease; NIUA, NSAID-induced urticaria-angioedema without underlying chronic diseases; NSAID, nonsteroidal anti-inflammatory drugs; PRC, paracetamol; and SNIUAA, Single-NSAID-induced urticaria/angioedema or anaphylaxis.

**Table 2:** Comparison of group A1 with group A2 in terms of clinical and demographic characteristics.

	Group A2 (Positive Reexposure)	Group A1 (Negative Reexposure)	*p*-Value
age (y)	55.8 ± 11.1	52.2 ± 1.6	ns
time span to interview (m)	36.5 ± 19.7	32.8 ± 18.0	ns
sex: F/M (*n*)	5/7	98/21	0.001
NIUA (%)	25%	58%	ns
SNIUAA (%)	58%	40%	ns
NERD (%)	17%	2%	ns
any positive SPT for seasonal allergens (%)	14%	25%	ns
any positive SPT for perennial allergens (%)	43%	27%	ns

y, years; m, months; SPT, skin prick test, *n*, number of patients; age and time span are presented as mean ± SD.

**Table 3:** Comparison of group A with group B in terms of clinical and demographic characteristics.

	Group A (*n* = 131) The Reexposure Took Place	Group B (*n* = 33) No Reexposure Took Place	*p*-Value
age (y)	52.5 ± 16.2	54.2 ± 15.8	ns
time span to interview (m)	33.1 ± 18.1	30.9 ± 17.9	ns
sex: F/M (*n*)	103/28	27/6	ns
NIUA (%)	54%	52%	ns
SNIUAA (%)	42%	45%	ns
NERD (%)	4%	3%	ns
any positive SPT for seasonal allergens (%)	24%	9%	ns
any positive SPT for perennial allergens (%)	29%	25%	ns

y, years; m, months; SPT, skin prick test, *n*, number of patients; age and time span are presented as mean ± SD.

**Table 4:** Excipients for which the search revealed data on hypersensitivity reactions or immune responses related to them, together with the number of recorded publications.

Substance	Number of Records	Hypersensitivity Reaction or Immune-Mediate Response to a Given Substance	References
Acesulfame potassium	2	hives and discomfort in the throat, swelling of the lips and face, sinus congestion, and difficulty breathing	[31]
Alpha-tocopherol	5	contact dermatitis	[32]
Aspartame	5	urticaria	[33]
Carmine	30	anaphylaxis, dye-induced immediate allergy	[34,35]
Calcium phosphate	4	positive patch test, contact dermatitis	[36]
Cetyl alcohol	5	contact dermatitis	[37]
Citric acid	7	anaphylaxis	[38]
Cochineal red	2	anaphylaxis	[39]
Colloidal silica	1	skin hypersensitivity	[40]
Corn starch	1	cell-mediated immunity	[41]
Croscarmellose sodium	1	erythematous skin rash with diffuse itching	[42]
Dimethicone	1	contact dermatitis	[43]
Glycine	18	anaphylaxis	[44]
Gelatine	6	anaphylaxis	[45]
Hydrogenated castor oil	1	contact dermatitis	[46]
Hydroxypropyl cellulose	1	cross-reactivity to propylene glycol, contact dermatitis	[47]
Hypromellose	3	contact dermatitis, anaphylaxis	[48,49]
Macrogol	14	anaphylaxis	[50]
Maltodextrin	1	sterile peritonitis, delayed reaction	[51]
Mannitol	19	anaphylaxis	[52,53,54,55]
Polysorbate 80	19	anaphylaxis, urticaria	[56,57]
Polyvinylpyrrolidone	6	anaphylaxis	[58,59]
Povidone	41	anaphylaxis	[60,61,62]
Potato starch	1	anaphylaxis	[63]
Propylene glycol	20	immediate drug hypersensitivity reactions	[64]
Quinoline yellow	3	fixed food and drug-induced eruption	[65]
Sodium benzoate	2	pruritus, fixed drug eruption	[66,67]
Sorbic acid	2	generalized contact urticaria	[68]
Sorbitol	7	allergic contact dermatitis	[69]
Starch	28	anaphylaxis	[70]
Stearic acid	5	cosmetic allergy from stearic acid and glyceryl stearate	[71]
Sunset yellow	3	oro-facial granulomatosis, eczema	[72,73]
Titanium dioxide	7	allergic contact dermatitis	[74]

## Data Availability

Datasets analyzed in this study are available from the corresponding author upon reasonable request.

## Supplementary Materials

The following supporting information can be downloaded at: https://www.mdpi.com/article/10.3390/diagnostics12123074/s1, Table S1: Medicinal products registered in Poland and containing acetylsalicylic acid as an active pharmaceutical ingredient together with the corresponding excipients, divided into those for which the search revealed or did not reveal data on related to them hypersensitivity reaction or immune response; Table S2: Medicinal products registered in Poland and containing acetaminophen as an active pharmaceutical ingredient together with the corresponding excipients, divided into those for which the search revealed or did not reveal data on related to them hypersensitivity reaction or immune response; Table S3: Medicinal products registered in Poland and containing meloxicam as an active pharmaceutical ingredient together with the corresponding excipients, divided into those for which the search revealed or did not reveal data on related to them hypersensitivity reaction or immune response; Table S4: Medicinal products registered in Poland and containing diclofenac as an active pharmaceutical ingredient together with the corresponding excipients, divided into those for which the search revealed or did not reveal data on related to them hypersensitivity reaction or immune response; and Figure S1: Comparison of the similarity of the structures of the exemplary analgesics and excipients. Chemical formulas were created with the ACD/ChemSketch software, 1 February 2018.
