# Supplementary material for: Diagnostic Value of Oral Provocation Tests in Drug Hypersensitivity Reactions Induced by Nonsteroidal Anti-Inflammatory Drugs and Paracetamol
Source: Diagnostics (Basel). 2022 Dec 7;12(12):3074. doi: 10.3390/diagnostics12123074 (PMC9777020; doi:10.3390/diagnostics12123074)

diagnostics

Supplementary Data

Table S1. Medicinal products registered in Poland and containing acetylsalicylic acid as an active pharmaceutical ingredient together with the corresponding excipients, divided into those for which the search revealed or do not revealed data on related to them hypersensitivity reaction or immune response.

Pharmaceutical form	Medicinal product	Excipients	
		with reported hypersensitivity	without data on hypersensitivity
Effervescent tablets	**Alka-Prim**	Citric acid, Glycine	Sodium bicarbonate
Effervescent tablets	**Alka-Seltzer**	Citric acid, Povidone, Sodium benzoate	Calcium silicate, Dimethicone, Lemon flavor, Lime flavor, Lime flavor, Sodium bicarbonate, Sodium docusan, Sodium saccharin
Effervescent tablets	**Aspirin musująca**	Citric acid	Sodium bicarbonate, Sodium carbonate, Sodium citrate
Effervescent tablets	**Ultrapiryna Fast**	Citric acid, Mannitol, Povidone	Simethicone, Sodium bicarbonate, Sodium carbonate, Sodium citrate, Sodium dihydrogen citrate, Sodium docusate
Enteric-coated tablets	**Abrea**	Carmine, Colloidal silica, Macrogol, Polysorbate 80, Potato starch, Sunset yellow, Titanium dioxide	Aluminum lake, Lactose monohydrate, Methacrylic acid and ethyl acrylate copolymer, Microcrystalline cellulose, Polyvinyl alcohol, Sodium dodecyl sulfonate, Talc, Triacetin
Enteric-coated tablets	**Acard**	Colloidal silica, Corn starch, Hypromellose, Titanium dioxide	Acryleze, Anti-foaming emulsion, Methacrylic acid copolymer, Powdered cellulose, Sodium bicarbonate, Sodium lauryl sulfate, Talc, Triethyl citrate
Enteric-coated tablets	**Acecardin**	Colloidal silica, Hypromellose, Macrogol, Gelatinized starch, Stearic acid, Titanium dioxide,	Lactose monohydrate, Methacrylic acid copolymer, Microcrystalline cellulose, Powdered cellulose, Sodium bicarbonate, Sodium lauryl sulfate, Talc, Triacetin
Enteric-coated tablets	**Acetylsalicylic Acid Sandoz**	Colloidal silica, Corn starch, Polysorbate 80, Stearic acid	Methacrylic acid and ethyl acrylate copolymer, Microcrystalline cellulose, Sodium lauryl sulfate, Talc, Triethyl citrate
Enteric-coated tablets	**Acoren**	Colloidal silica, Corn starch, Hypromellose, Stearic acid, Titanium dioxide	Methacrylic acid and ethyl acrylate copolymer, Microcrystalline cellulose, Sodium bicarbonate, Sodium carboxymethyl starch, Sodium lauryl sulfate, Talc, Triacetin, Triethyl citrate
Enteric-coated tablets	**Alepton**	Carmine, Colloidal silica, Macrogol, Potato starch, Sunset yellow Titanium dioxide	Aluminum lake, Lactose monohydrate, Methacrylic acid and ethyl acrylate copolymer, Microcrystalline cellulose, Opadry ii yellow, Polyvinyl alcohol, Pomry II pink, Talc, Triacetin
Enteric-coated tablets	**Anacard medica protect**	Colloidal silica, Hypromellose, Gelatinized starch, Titanium dioxide	Acryleze, Fallout ys-1-7027 white, Methacrylic acid and ethyl acrylate copolymer, Microcrystalline cellulose, Sodium bicarbonate, Sodium lauryl sulfate, Talc, Triacetin, Triethyl citrate

Enteric-coated tablets	**Asapirine 150 mg**	Colloidal silica, Corn starch, Hypromellose, Stearic acid, Titanium dioxide	Methacrylic acid and ethyl acrylate copolymer, Microcrystalline cellulose, Sodium bicarbonate, Sodium lauryl sulfate, Talc, Triacetin, Triethyl citrate
Enteric-coated tablets	**Aspicont**	Colloidal silica, Corn starch, Hypromellose, Stearic acid, Titanium dioxide	Methacrylic acid and ethyl acrylate copolymer, Microcrystalline cellulose, Sodium bicarbonate, Sodium lauryl sulfate, Talc, Triacetin, Triethyl citrate
Enteric-coated tablets	**Bonacard**	Colloidal silica, Hypromellose, Gelatinized starch, Macrogol, Stearic acid, Titanium dioxide	Lactose monohydrate, Methacrylic acid copolymer, Microcrystalline cellulose, Powdered cellulose, Sodium bicarbonate, Sodium lauryl sulfate, Talc, Triacetin
Enteric-coated tablets	**Cardiopirin**	Colloidal silica, Corn starch, Polysorbate 80, Stearic acid	Methacrylic acid and ethyl acrylate copolymer, Microcrystalline cellulose, Sodium lauryl sulfate, Talc, Triethyl citrate
Enteric-coated tablets	**Cardioteva**	Colloidal silica, Corn starch, Polysorbate 80, Stearic acid	Methacrylic acid and ethyl acrylate copolymer, Microcrystalline cellulose, Sodium lauryl sulfate, Talc, Triethyl citrate
Enteric-coated tablets	**Encopirin Cardio 81**	Colloidal silica, Potato starch, Titanium dioxide	Aqua polish white, Aqua polish, Microcrystalline cellulose, Talc
Enteric-coated tablets	**Etopiryna PRO**	Cochineal red, Colloidal silica, Corn starch, Hypromellose, Titanium dioxide	Methacrylic acid copolymer, Sodium bicarbonate, Sodium lauryl sulfate, Talc, Triethyl citrate
Enteric-coated tablets	**Eubiocard**	Colloidal silica, Hypromellose, Gelatinized starch, Macrogol, Stearic acid, Titanium dioxide	Lactose monohydrate, Methacrylic acid copolymer, Microcrystalline cellulose, Powdered cellulose, Sodium bicarbonate, Sodium lauryl sulfate, Talc, Triacetin
Enteric-coated tablets	**Lecardi**	Hydroxypropyl cellulose, Hypromellose, Gelatinized starch, Titanium dioxide	Aqua polish clear, Aqua polish, Carmellose sodium, Hydrogenated cottonseed oil, Methacrylic acid and ethyl acrylate copolymer, Microcrystalline cellulose, Talc, Triethyl citrate
Enteric-coated tablets	**Polocard**	Cochineal red, Colloidal silica, Corn starch, Hypromellose, Titanium dioxide	Methacrylic acid copolymer, Powdered cellulose, Sodium bicarbonate, Sodium lauryl sulfate, Talc, Triethyl citrate
Enteric-coated tablets	**Polopiryna Max**	Cochineal red, Colloidal silica, Corn starch, Hypromellose, Titanium dioxide	Methacrylic acid copolymer, Sodium bicarbonate, Sodium lauryl sulfate, Talc, Triethyl citrate
Enteric-coated tablets	**Proficar**	Corn starch, Povidone, Titanium dioxide	Iron oxide yellow, Methacrylic acid and ethyl acrylate copolymer, Microcrystalline cellulose, Talc, Triethyl citrate
Enteric-coated tablets	**Ultrapiryna**	Colloidal silica, Hypromellose, Titanium dioxide	Acryleze, Fallout ys-1-7027, Methacrylic acid copolymer, Microcrystalline cellulose, Sodium bicarbonate, Sodium lauryl sulfate, Talc, Triacetin, Triethyl citrate
Enteric-coated tablets	**Ultrapiryna Forte**	Corn starch, Povidone, Titanium dioxide	Iron oxide yellow, Methacrylic acid and ethyl acrylate copolymer, Microcrystalline cellulose, Talc, Triethyl citrate

Film coated tablets	**Aspirin 300**	Corn starch, Polysorbate 80	Methacrylic acid copolymer, Powdered cellulose, Sodium lauryl sulfate, Talc, Triethyl citrate
Film coated tablets	**Aspirin Cardio**	Corn starch, Polysorbate 80	Methacrylic acid copolymer, Sodium lauryl sulfate, Talc, Triethyl citrate
Film coated tablets	**Aspirin Pro**	Colloidal silica, Hypromellose	Sodium carbonate
Tablets	**Acard**	Corn starch	Microcrystalline cellulose, Powdered cellulose
Tablets	**Acesan**	Cochineal red, Stearic acid	Microcrystalline cellulose, Sodium carboxymethyl starch
Tablets	**Aspirin**	Corn starch	Cellulose
Tablets	**Bonapiryna**	Gelatinized starch, Stearic acid	Microcrystalline cellulose, Powdered cellulose
Tablets	**Eupirin**	Gelatinized starch, Stearic acid	Microcrystalline cellulose, Powdered cellulose
Tablets	**Gripblocker**	Corn starch	Microcrystalline cellulose, Powdered cellulose, Talc
Tablets	**Maxipirin**	Corn starch	Powdered cellulose
Tablets	**Polopiryna S**	Corn starch	Microcrystalline cellulose

Table S2. Medicinal products registered in Poland and containing acetaminophen as an active pharmaceutical ingredient together with the corresponding excipients, divided into those for which the search revealed or do not revealed data on related to them hypersensitivity reaction or immune response.

**Pharmaceutical form**	**Medicinal product**	**Excipients**	
		**with reported hypersensitivity**	**without data on hypersensitivity**
Tablets	**Paracetamol Accord**	Colloidal silica, Corn starch, Gelatine, Starch	Magnesium stearate, Talc
Tablets	**Paracetamol Aflofarm**	Corn starch, Gelatinized starch, Povidone, Stearic acid, Titanium dioxide	Crospovidone, Methacrylic acid and ethyl acrylate copolymer (1: 1), Microcrystalline cellulose, Talc
Tablets	**Paracetamol APTEO MED**	Potato starch, Povidone, Sorbitol, Starch	Magnesium stearate
Tablets	**Paracetamol Aurovitas**	Colloidal silica, Gelatinized starch, Hydroxypropyl cellulose	Magnesium stearate, Talc
Tablets	**Paracetamol Biofarm**	Croscarmellose sodium, Povidone, Gelatinized starch, Stearic acid	Crospovidone, Magnesium stearate, Microcrystalline cellulose
Tablets	**Paracetamol DOZ**	Colloidal silica, Corn starch, Povidone, Starch	Magnesium stearate
Tablets	**Paracetamol Filofarm**	Polyvinylpyrrolidone, Starch, Stearic acid	-
Tablets	**Paracetamol Polfa-Łódź**	Gelatinized starch, Povidone, Stearic acid	-
Tablets	**Paracetamol Synoptis**	Gelatinized starch, Povidone, Stearic acid	-

Tablets	**Paracetamol Teva**	Colloidal silica, Corn starch, Croscarmellose sodium, Povidone, Starch	Magnesium stearate, Microcrystalline cellulose, Talc
Tablets	**Paracetamolum Farmalider**	Gelatinized starch, Povidone, Stearic acid	Crospovidone, Magnesium stearate, Microcrystalline cellulose
Tablets	**Acenol**	Croscarmellose sodium, Povidone, Starch, Stearic acid	-
Tablets	**Apap**	Croscarmellose sodium, Hypromellose, Gelatinized starch, Macrogol, Povidone, Stearic acid	Carnauba wax
Tablets	**Codipar**	Colloidal silica, Potato starch, Povidone, Starch	Magnesium stearate, Microcrystalline cellulose, Talc
Tablets	**Laboratoria PolfaŁódź Paracetamol**	Potato starch, Povidone, Sorbitol, Starch	Magnesium stearate
Tablets	**Panacit**	Povidone, Gelatinized starch, Starch, Stearic acid	-
Tablets	**Panadol**	Corn starch, Gelatinized starch, Hypromellose, Povidone, Starch, Stearic acid	Carnauba wax, , Potassium sorbate, Triacetin
Tablets	**Paramax Rapid 500 mg**	Povidone, Starch, Stearic acid	Magnesium stearate, Microcrystalline cellulose
Effervescent tablets	**Paracetamol Aristo**	Citric acid, Maltodextrin, Povidone, Sodium benzoate, Sorbitol	Copovidone, Lemon flavor, Sodium bicarbonicum, Sodium carbonate, Sodium cyclamate, Sodium dihydrogen citrate, Sodium docusate, Sodium saccharin
Effervescent tablets	**Efferalgan**	Citric acid, Povidone, Sodium benzoate, Sorbitol,	Sodium bicarbonicum, Sodium carbonate, Sodium docusate, Sodium saccharin
Effervescent tablets	**Megapar**	Alpha-tocopherol, Citric acid, Macrogol, Maltodextrin, Povidone, Sorbitol	Arabic gum, Corn maltodextrin, Lemon flavor, Simethicone, Sodium bicarbonicum, Sodium carbonate, Sodium saccharin
Film coated tablets	**Paracetamol Biofarm**	Croscarmellose sodium, Gelatinized starch, Hypromellose, Povidone, Stearic acid	Aquapolish clear, Crospovidone, Glycerol, Magnesium stearate, Talc
Film coated tablets	**Paracetamol Hasco**	Corn starch, Hypromellose, Maltodextrin, Povidone, Starch, Titanium dioxide	Magnesium stearate, Polydextrose, Potassium sorbate, Talc, Triglycerides of medium chain saturated fatty acids
Film coated tablets	**Panadol Sprint**	Colloidal silica, Hypromellose, Gelatinized starch, Macrogol, Polysorbate 80, Povidone, Starch, Titanium dioxide	Alginic acid, Calcium carbonate, Carnauba wax, Crospovidone, Magnesium stearate, Opadry white
Film coated tablets	**Panaprex**	Colloidal silica, Croscarmellose sodium,	Methacrylic acid and ethyl acrylate copolymer (1: 1), Polyvinyl alcohol, Sodium carbonate, Sodium stearyl fumarate, Talc

		Macrogol, Povidone, Titanium dioxide	

Table S3. Medicinal products registered in Poland and containing meloxicam as an active pharmaceutical ingredient together with the corresponding excipients, divided into those for which the search revealed or do not revealed data on related to them hypersensitivity reaction or immune response.

Pharmaceutical form	Medicinal product	Excipients	
		**with reported hypersensitivity**	**without data on hypersensitivity**
Tablets	**Aglan 15**	Colloidal silica	Crospovidone, Lactose monohydrate, Magnesium stearate, Microcrystalline cellulose, Sodium Citrate
Tablets	**Aspicam**	Povidone	Crospovidone, Lactose monohydrate, Magnesium stearate
Tablets	**Lormed 15**	Colloidal silica, Corn starch	Lactose monohydrate, Magnesium stearate, Microcrystalline cellulose, Pregelatinized starch, Sodium Citrate
Tablets	**Melobax 15**	Colloidal silica, Corn starch	Lactose monohydrate, Magnesium stearate, Microcrystalline cellulose, Pregelatinized starch, Sodium Citrate
Tablets	**Meloksam**	Colloidal silica	Crospovidone, Lactose monohydrate, Magnesium stearate, Microcrystalline cellulose, Sodium Citrate
Tablets	**Meloxic**	Colloidal silica, Corn starch	Lactose monohydrate, Magnesium stearate, Microcrystalline cellulose, Sodium Citrate
Tablets	**Meloxicam Adamed**	Colloidal silica, Povidone	Crospovidone, Lactose monohydrate, Magnesium stearate, Microcrystalline cellulose, Sodium Citrate
Tablets	**MeloxiMed**	Colloidal silica	Crospovidone, Lactose monohydrate, Magnesium stearate, Microcrystalline cellulose, Sodium Citrate
Tablets	**MeloxiMed Forte**	Colloidal silica	Crospovidone, Lactose monohydrate, Magnesium stearate, Microcrystalline cellulose, Sodium Citrate
Tablets	**Meloxistad**	Colloidal silica, Corn starch	Lactose monohydrate, Magnesium stearate, Microcrystalline cellulose, Pregelatinized starch, Sodium Citrate
Tablets	**Moilec**	Colloidal silica,	Crospovidone, Lactose monohydrate, Magnesium stearate, Microcrystalline cellulose, Sodium Citrate
Tablets	**Movalis**	Colloidal silica, Povidone	Crospovidone, Lactose monohydrate, Magnesium stearate, Microcrystalline cellulose, Sodium Citrate
Tablets	**Noflamen**	Colloidal silica, Povidone	Crospovidone, Lactose monohydrate, Magnesium stearate, Microcrystalline cellulose, Sodium Citrate
Tablets	**Opokan**	Colloidal silica, Corn starch	Lactose monohydrate, Magnesium stearate, Microcrystalline cellulose, Pregelatinized starch, Sodium Citrate
Tablets	**Remolexam**	Colloidal silica, Povidone	Crospovidone, Lactose monohydrate, Magnesium stearate, Microcrystalline cellulose, Sodium Citrate
Tablets	**Reumelox**	Colloidal silica, Corn starch	Lactose monohydrate, Magnesium stearate, Microcrystalline cellulose, Sodium Citrate
Orally disintegrating tablets (ODT)	**Mel**	Acesulfame potassium, Aspartame, Corn starch, Mannitol, Povidone	Betadex, Crospovidone, Ludiflash, Pearlitol Flash, Polyvinyl acetate, Raspberry flavor SD0621M, Sodium Citrate, Sodium stearyl fumarate, Vanilla flavor SD1333

Orally disintegrating tablets (ODT)	**Trosicam**	Aspartame, Citric acid, Mannitol, Povidone, Sorbitol	Crospovidone, Forest fruit aroma, Magnesium stearate, Sodium lauryl sulfate, Talc, Yoghurt flavor

Table S1. Medicinal products registered in Poland and containing diclofenac as an active pharmaceutical ingredient together with the corresponding excipients, divided into those for which the search revealed or do not revealed data on related to them hypersensitivity reaction or immune response.

**Pharmaceutical form**	**Medicinal product**	**Excipients**	
		**with reported hypersensitivity**	**without data on hypersensitivity**
Enteric-coated tablets	**Diclac 50**	Colloidal silica, Corn starch, Titanium dioxide	Calcium hydrogen phosphate dihydrate, Eudragit, Iron oxide yellow, Lactose monohydrate, Magnesium stearate, Microcrystalline cellulose, Sodium carboxymethyl starch, Talc, Triethyl citrate
Enteric-coated tablets	**Majamil PPH**	Corn starch, Dimethicone, Polysorbate 80, Sorbic acid, Titanium dioxide	Gelatinized starch, Iron oxide red, Iron oxide yellow, Lactose monohydrate, Macrogol, Magnesium stearate, Methacrylic acid and ethyl acrylate copolymer, Microcrystalline cellulose, Sodium carboxymethyl starch, Talc
Enteric-coated tablets	**Voltaren**	Colloidal silica, Corn starch, Hypromellose, Povidone, Titanium dioxide	Anti-foaming emulsion, Glyceryl hydroxystearate, Iron oxide red, Iron oxide yellow, Lactose monohydrate, Macrogol, Magnesium stearate, Methacrylic acid and ethyl acrylate copolymer, Microcrystalline cellulose, Sodium carboxymethyl starch, Talc
Prolonged release tablets	**Diclac 150 Duo**	Colloidal silica, Corn starch, Hypromellose	Calcium hydrogen phosphate dihydrate, Iron oxide red, Lactose monohydrate, Magnesium stearate, Microcrystalline cellulose, Sodium carboxymethyl starch
Prolonged release tablets	**Dicloreum retard**	Hydroxypropyl cellulose, Povidone, Titanium dioxide	Diethyl phthalate, Ethyl cellulose, Magnesium stearate, Talc
Prolonged release tablets	**Majamil prolongatum**	Colloidal silica, Hypromellose, Povidone, Propylene glycol, Sunset yellow, Titanium dioxide	Cellulose, powder, Eudragit, Macrogol, Magnesium stearate, Talc
Prolonged release tablets	**Voltaren SR 100**	Cetyl alcohol, Colloidal silica, Hypromellose, Polysorbate 80, Povidone, Titanium dioxide	Iron oxide red, Macrogol, Magnesium stearate, Saccharose, Talc
Film coated tablets	**Cataflam 50**	Calcium phosphate, Colloidal silica, Corn starch, Povidone, Titanium dioxide	Iron oxide red, Macrogol, Magnesium stearate, Saccharose, Sodium carboxymethyl starch, Talc
Film coated tablets	**Diclomax**	Colloidal silica, Croscarmellose sodium, Titanium dioxide	Calcium hydrogen phosphate dihydrate, Gelatinized starch, Iron oxide black, Iron oxide red, Iron oxide yellow, Macrogol,

			Magnesium stearate, Polyvinyl alcohol, Pregelatinized corn starch, Talc
Film coated tablets	**Dicloreum**	Povidone, Titanium dioxide	Cellulose acetate phthalate, Diethyl phthalate, Gelatinized starch, Lactose monohydrate, Magnesium stearate
Film coated tablets	**Dicuno**	Cochineal red, Colloidal silica, Croscarmellose sodium, Titanium dioxide	Calcium hydrogen phosphate dihydrate, Gelatinized starch, Iron oxide red, Iron oxide yellow, Macrogol, Magnesium stearate, Polyvinyl alcohol, Pregelatinized corn starch, Talc
Film coated tablets	**Olfen 25**	Colloidal silica, Hypromellose, Quinoline yellow, Titanium dioxide	Iron oxide yellow, Macrogol, Methacrylic acid and ethyl acrylate copolymer, Sodium carboxymethyl starch, Sodium stearyl fumarate, Talc, Triethyl citrate
Film coated tablets	**Voltaren Acti**	Colloidal silica, Corn starch, Hypromellose, Povidone, Stearic acid, Titanium dioxide	Lactose monohydrate, Magnesium stearate, Sodium carboxymethyl starch
Film coated tablets with prolonged release	**Olfen 75 SR**	Hypromellose, Titanium dioxide	Iron oxide red, Lactose monohydrate, Macrogol, Magnesium stearate, Talc
Film coated tablets with prolonged release	**Olfen UNO**	Colloidal silica, Hydrogenated castor oil, Hypromellose, Mannitol	Ethyl cellulose, Iron oxide yellow, Magnesium stearate, Sodium starch gliconate, Talc

Figure S1. Comparison of the similarity of the structures of the exemplary analgesics and excipients. Chemical formulas were created with the ChemSketch software, 2018.2.1. (Advanced Chemistry Development Inc., Ontario, Canada).

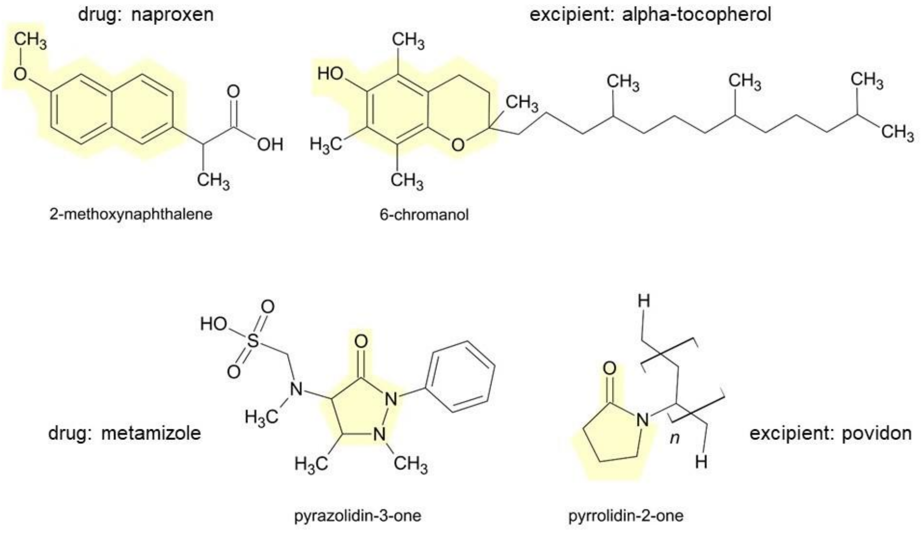

Supplement: Supplementary file 1 [file diagnostics-12-03074-s001.zip › diagnostics-2002433-supplementary.pdf]
